# Comparative *in Vivo* Assessment of Some Adverse Bioeffects of Equidimensional Gold and Silver Nanoparticles and the Attenuation of Nanosilver’s Effects with a Complex of Innocuous Bioprotectors

**DOI:** 10.3390/ijms14022449

**Published:** 2013-01-25

**Authors:** Boris A. Katsnelson, Larisa I. Privalova, Vladimir B. Gurvich, Oleg H. Makeyev, Vladimir Ya. Shur, Yakov B. Beikin, Marina P. Sutunkova, Ekaterina P. Kireyeva, Ilzira A. Minigalieva, Nadezhda V. Loginova, Marina S. Vasilyeva, Artem V. Korotkov, Eugene A. Shuman, Larisa A. Vlasova, Ekaterina V. Shishkina, Anastasia E. Tyurnina, Roman V. Kozin, Irene E. Valamina, Svetlana V. Pichugova, Ludmila G. Tulakina

**Affiliations:** 1 The Medical Research Center for Prophylaxis and Health Protection in Industrial Workers, 30 Popov Str., Ekaterinburg 620014, Russia; E-Mails: privalovali@yahoo.com (L.I.P.); gurvich@ymrc.ru (V.B.G.); marinasutunkova@yandex.ru (M.P.S.); katerinakir@yandex.ru (E.P.K.); ilzira-minigalieva@yandex.ru (I.A.M.); tushkanN@yandex.ru (N.V.L.); 2 The Ural State Medical Academy, 17 Klyuchevskaya Str., Ekaterinburg 620109, Russia; E-Mails: ommt305@mail.ru (O.H.M.); myshkalev@rambler.ru (M.S.V.); akorotkov64@mail.ru (A.V.K.); eshuman@gmail.com (E.A.S.); larus-a@rambler.ru (L.A.V.); valamina2012@yandex.ru (I.E.V.); 3 The Institute of Natural Sciences, the Ural Federal University, Ekaterinburg 620000, Russia; E-Mails: vladimir.shur@usu.ru (V.Y.S.); ekaterina.shishkina@labfer.usu.ru (E.V.S.); anastasiya.tyurnina@labfer.usu.ru (A.E.T.); roman.kozin@labfer.usu.ru (R.V.K.); 4 The City Clinical Diagnostics Centre, 38 Dekabristov Str., Ekaterinburg 620142, Russia; E-Mails: kdc_boss@mail.ru (Y.B.B.); ekb-lem@mail.ru (S.V.P.); tulakina@inbox.ru (L.G.T.)

**Keywords:** nanosilver, nanogold, bronchoalveolar lavage, subchronic toxicity, genotoxicity, bioprotectors

## Abstract

Stable suspensions of nanogold (NG) and nanosilver (NS) with mean particle diameter 50 and 49 nm, respectively, were prepared by laser ablation of metals in water. To assess rat’s pulmonary phagocytosis response to a single intratracheal instillation of these suspensions, we used optical, transmission electron, and semi-contact atomic force microscopy. NG and NS were also repeatedly injected intraperitoneally into rats at a dose of 10 mg/kg (0.5 mg per mL of deionized water) three times a week, up to 20 injections. A group of rats was thus injected with NS after oral administration of a “bioprotective complex” (BPC) comprised of pectin, multivitamins, some amino acids, calcium, selenium, and omega-3 PUFA. After the termination of the injections, many functional and biochemical indices and histopathological features of the spleen, kidneys and liver were evaluated for signs of toxicity, and accumulation of NG or NS in these organs was measured. From the same rats, we obtained cell suspensions of different tissues for performing the RAPD test. It was demonstrated that, although both nanometals were adversely bioactive in all respects considered in this study, NS was more noxious as compared with NG, and that the BPC tested by us attenuated both the toxicity and genotoxicity of NS.

## 1. Introduction

The ever-increasing number of studies devoted to various aspects of the biological activity of nanoparticles (NPs), including their toxicity, is almost on a par with the pace of developments in the area of nanotechnologies. This inspires hope that a sufficiently reliable evidence base will be obtained in the foreseeable future for assessing health risks, not only associated with the production and broad use of nanomaterials, but also with the contribution of NPs (“ultrafine particles”) to the aerosol contamination of the ambient and workplace air in many traditional industries. Consequently, our team has focused on the effects of metal and metal-oxide NPs, samples of which, with reliably preset dimensional and other relevant characteristics, could be regarded as a model for studying general patterns in the adverse influence on the organism, not only of respective engineered nanomaterials, but also of the ultrafine fraction of “usual” aerosol air pollutants.

Our earlier studies [1–6] showed, with reference to iron oxide Fe_3_O_4_ (magnetite), that, given equal doses by mass, NPs feature considerably higher cytotoxicity for lung macrophages and higher subchronic systemic toxicity than micrometric particles of the same chemical composition while triggering a more active protective response of pulmonary (alveolar) phagocytosis. At the same time, it was demonstrated that, within the conventional nanometric range, the relationship between particle diameter and toxicity is intricate and non-unique, which may be due to differences in the toxicokinetics, which is controlled by both unequally active physiological mechanisms responsible for the elimination and retention of NPs of various diameters, their unequal penetrability through biological barriers and, finally, their unequal solubility.

Among the issues that need to be resolved for developing both the theoretical foundations of comparative nanotoxicology and its regulatory aspects, of paramount importance is the question of which nanomaterial characteristics play the most important role:

1. Those associated with the nano-dimension of particles of any chemical composition (*i.e.*, a high probability of NP deposition in the naso-pharyngeal region and lower airways; an ability to penetrate through biological barriers, transport with the lymph and blood to remote organs and the retention in them; the penetration into cells and cellular organelles; a large specific surface area; the peculiar character of physical interactions and chemical processes to which the mechanisms of damaging impact at subcellular and cellular levels are related); or,
2. The chemical nature of the NP-forming substance, which defines important mechanisms of its toxic impact both in the ionic-molecular form and in the form of particles of various sizes.

Within the framework of this problem, we set ourselves the task of comparing the toxic effects of virtually equidimensional silver and gold NPs. The choice of these two metals was determined not only by the theoretical premises set out above, but also by the fact that, in bulk, they are almost inert biologically. Furthermore, the high practical importance of nanosilver (NS) and nanogold (NG), have partly overlapping applications.

A great number of studies have been devoted to assessing the toxicity of various NS samples (e.g., [7–17]). A comparison of the results obtained from these studies with the data of the fewer works devoted to the assessment of similar effects evoked by NG (e.g., [18–26]) have suggested that the latter is far less toxic, although we are aware of only some studies in which such a comparison was really carried out in parallel testing conditions, and even those studies were performed not on mammals but on *Daphnia magna* [27] or zebrafish (*Danio rerio*) embryos [28].

Special consideration should be given to the issue of possible genotoxicity of even those nanomaterials that do not feature it in an ion-molecular state or in the form of micrometric particles. Recently, experimental evidence has been obtained, confirming that various nanomaterials are capable of producing damaging effects on DNA—namely, NPs of titanium dioxide [12,29,30], zinc oxide [31], silicon dioxide [32,33], carbon black [34], *etc.* It should be noted, however, that not all researchers have received positive results for the same nanomaterials, particularly when testing them *in vivo* rather than on cell lines (for example, for nano-titanium dioxide in the crystal form of anatase [35]).

A large number of papers have been published demonstrating, by means of various tests and on various test objects, the genotoxic effect of nanosilver, again, however, these tests were predominantly *in vitro* (e.g., [14,32,36–44]). A small number of studies using short-term *in vivo* tests quite often yielded a negative result (e.g., [41]). Many authors emphasize the role of the NP-stimulated oxidative stress in the mechanisms of NPs’ damaging effect on DNA. Indeed, some studies not only have shown that NS increases considerably the formation of reactive oxygen species (ROS), but also have revealed significant inhibition of the genotoxic effects of NS in the presence of antioxidants or free radical scavengers [32,39,43,45].

There is less information concerning the genotoxicity of nanogold, and it is rather contradictory. Some data indicates that NG causes genome instability, also through oxidative stress [46], but other experimenters have established that neither nano- (2 nm or 20 nm), nor microparticles (200 nm) of gold possess genotoxicity either *in vitro* or in rats exposed to three consecutive intravenous [23] or a single intratracheal [47] administration.

We have found only one paper in which the genotoxicity of NG and NS was compared in parallel [48]. The authors established on a human hepatocellular carcinoma cell line (HepG2) that at concentrations (by metal) of up to 100 μM NS provokes greater DNA damage than NG.

On the whole, a review of the above sources, and some additional ones, suggests that NG is not only generally less toxic but also possesses lower genotoxicity as compared with NS. Having stated this, however, we should qualify that:

- In too few works has it been possible to find direct comparison data for NS and NG toxicities in parallel testing using any experimental model and equidimensional nanoparticles of these metals under similar conditions of exposure. Moreover, we have not discovered any single chronic or, at least, subchronic, comparative experiment with NG and NS on laboratory mammals in the literature known to us.
- We also have not found any papers comparing the phagocytosis response to the pulmonary deposition of nanoparticles of these two metals, this response playing, as is known, a key role in pulmonary clearance. Our own pilot experiments involving intratracheal instillations of NG and NS suspensions have shown that NS provokes a substantially greater increase in the cell count of the bronchoalveolar lavage fluid (BALF), and, judging from the ratio of neutrophil leukocytes to alveolar macrophages in BALF, NS is more cytotoxic for alveolar macrophages than NG [5]. These newly obtained data, however, need to be reproduced, preferably in experiments with the same NG and NS samples that would be used for comparative assessment of systemic toxicity and genotoxicity.
- We have found no information concerning any attempts to enhance the resistance of the whole organism to systemic toxic and genotoxic impacts of these nanomaterials, although the above data on the protective action *in vitro* of certain antioxidant and antiradical substances point directly to one of the possible approaches to such “biological prophylaxis.” The meaning of this concept, general principles of bioprophylaxis and numerous examples of their realization have been described by us repeatedly, including in review articles (e.g., [49]). Beyond any doubt, ours were not the only studies demonstrating a possibility to reduce some metals’ toxicity with this or that innocuous antagonist. For instance, it was recently shown that hepatic copper retention in ram lambs could be reduced by dietary supplementation with molybdenum and zinc [50]; or that quercetin and especially quercetin in combination with arginine ameliorated nano-zinc oxide’s nephrotoxicity for rats [51]. Significant reducing of the lead blood levels with the help of calcium dietary supplementation was demonstrated not only in a number of animal experiments, ours included, but also on pregnant women [52]. An important distinguishing feature of our approach is that we use multicomponent complexes of agents with different mechanisms of protective action rather than isolated bioprotectors [49].

We decided that the first attempt to ensure such protective effect on the organism should be undertaken in response to the effect of the more toxic of two nanometals under study, which is, presumably, the NS.

## 2. Results and Discussion

### 2.1. Pulmonary Phagocytosis Response to a Single Intratracheal Instillation

#### 2.1.1. Optical Microscopy Data

Table 1 presents the results of a comparative estimation of shifts in the BALF cell population in response to intratracheal instillation of gold NPs and silver nano- or microparticles carried out within the framework of the present study, in comparison with the earlier published results of a similar experiment with finer NPs of the same metals [5]. Rather than in parallel, these two experiments were carried out in different periods (2011 and 2012) and—what is especially important—with different background BALF cell counts (as can be seen from Table 1, all indices for control rats were higher in the second experiment as compared with the first one). At the same time, it was shown within each experiment that:

- nanoparticles of both metals cause a substantial increase in the number of neutrophil leukocytes (NL) compared with an insignificant, or even unobservable, increase in the number of alveolar macrophages (AM) in the BALF and, thus, a sharp increase in the NL/AM ratio;
- for each of the nano-sizes tested, this ratio is considerably and statistically significantly higher for NS as compared with NG.

Along with the above, it was shown in the second experiment that the administration of silver microparticles results in a considerably and statistically lower NL/AM ratio than in response to nanoparticles of the same metal.

The recruitment of phagocytizing cells into the lower airways, manifesting itself in an increased number of BALF cells, is a typical reaction to the deposition of particles in them. As both total cell count and shift towards polymorphonuclear (mainly neutrophil) leukocytes (NL) become more marked, the damaging action of cytotoxic particles on alveolar macrophages (AM) increases [53–59]. The dependence of both indices on the number of destroyed AMs was experimentally modeled by: (1) the intratracheal instillation of aseptically obtained peritoneal macrophages that were destroyed (without the pre-incubation of these cells with any particles) by repeat freezing/thawing, or by ultrasonication, and (2) lipids extracted from these macrophage breakdown products. Such a dose-dependent imitation of the pattern of phagocyte recruitment toward cytotoxic particles with products of macrophage breakdown, on the one hand, and the good rank correlation (demonstrated in the same studies) between the aforementioned NL/AM shift and *in vitro* estimates (with the trypan blue exclusion test) of the capacity of different particulates to damage cultured peritoneal macrophage, on the other hand, justifies the usage of NL/AM ratio as a circumstantial, but rather informative, index of comparative cytotoxicity of particles *in vivo.*

As is well known, NL recruitment towards the free surface of lower airways in response to the deposition of particles, NPs included, is quite often described as “inflammation” and, thus, as a pathological phenomenon. We maintain, however, that this concept can be somewhat misleading. Beyond any doubt, enhanced recruitment of NLs is typical of acute and, to a lesser degree, chronic inflammatory processes induced by microbial or chemical agents. However, a certain number of these cells are always present in the BALF of healthy animals, at least so when they are constantly inhaling unfiltered air—are they really always living with chronic inflammation of the respiratory system?

In the meantime, there are fairly strong reasons for considering the response under consideration to be an important mechanism of partial compensation for the damage caused by cytotoxic particles to the alveolar macrophage, the main effector of pulmonary clearance. A mathematical multicompartmental model of pulmonary region clearance which describes just this compensatory mechanism simulates very well the retention of dusts of varying degrees of cytotoxicity (titanium dioxide, quartzite rock, standard quartz DQ12) in the lungs at long-term inhalation, and a decrease in this retention under the effect of a potent protector of the macrophage against the cytotoxicity of particles such as glutamate [57–59].

In our earlier experiments with Fe_3_O_4_ (magnetite) nanoparticles [1], we demonstrated that these particles are considerably more cytotoxic than chemically identical fine particles of the micrometer range (1 μm) as could be judged by the NL/AM ratio. The estimation being conducted in parallel, 10 nm particles were found to be considerably more cytotoxic compared with 50 nm particles. In this connection, it should not go unnoticed that, despite an essentially lower control (background) value for NL/AM in the 2011 experiment with nanosilver and nanogold compared with the 2012 experiment, this ratio was higher for both in the first case. In other words, an increase in the cytotoxicity with a decrease in the NP size is evident from comparison of these two experiments (with due reservation, that care should be exercised when comparing the data of different experiments if these are not carried out in parallel).

A comparison of nano- and microsilver confirms again that, given the same chemical nature of nano- and microparticles, the latter are much less cytotoxic and, consequently, provoke less intensive NL recruitment. At the same time, our experience shows that the number of AMs in BALF (reflecting a resultant of oppositely directed effects of their recruitment and destruction) at exposure to more cytotoxic particles often proves to be lower than at exposure to less cytotoxic ones; it is precisely this relationship that emerges when comparing nano- and microsilver. In other cases, however, the recruitment of new AM echelons (which, as well as the recruitment of NLs, is controlled by the mass of macrophage breakdown products but with another type of mathematical relationship between their dose and the number of recruited cells [53,54]) prevails over their destruction, so that the number of AMs proves higher under the action of more cytotoxic particles, as we can see in this experiment when comparing NS and NG.

In general, cytological analysis of BALF suggests the following:

- the deposition of gold and silver NPs (as well as of iron oxide NPs in our earlier studies) causes an active protective response of phagocyte recruitment, mainly that of neutrophils, this response being much stronger than that to microparticles of the same substance (as shown for silver and, earlier, for iron oxide);
- given the above reservation, there are grounds to assume that the cytotoxicity of both gold and silver grows with a reduction in particle size in the nanometric range (again similar to the dependence conclusively proven for iron oxide);
- given equal nanoparticle size, the cytotoxicity of NS as judged from the NL/AM ratio is considerably higher than that of NG.

#### 2.1.2. Semi-Contact Atomic Force Microscopy (sc-AFM) Data

As is well known, the starting point for the engulfment of a particle by a phagocytic cell (endocytosis) is a close contact between them, as a result of which a subjacent portion of the plasma membrane is as though indented (so-called invagination) and then pinched off forming a membrane-bound vesicle called endosome or phagosome. We proceeded from the assumption that the invagination process changes the topography of the phagocyte’s surface (be it an alveolar macrophage or a neutrophil leukocyte) leading to the formation of “pits,” and demonstrated in our experiments with nano- and microparticles of the iron oxide Fe_3_O_4_ (magnetite) that it was, indeed, a fact [1]. It was shown that both the quantity and the size of these pits depended on the predominant dimension of the particles being engulfed and on the phagocytic activity of the cell, which, in turn, depended on the particle’s cytotoxicity and thus, inversely, on its diameter again. This inverse dependence of phagocytic activity upon particle size was also demonstrated by counting internalized particles under optical microscopy [1,2].

Later on, the same phenomenon of pit formation was observed in our first comparative experiment with NS and NG suspensions involving particles of *ca.* 4 nm [5]. In that case, as the NPs under comparative study had virtually one and the same average diameter, pit dimensions proved independent of the chemical nature of NPs. However, the pits count per unit area of the cell’s surface was 1.5 times higher for NS than for NG. This seems to have shown again that more cytotoxic NPs are being engulfed more avidly.

**Figure 1:**
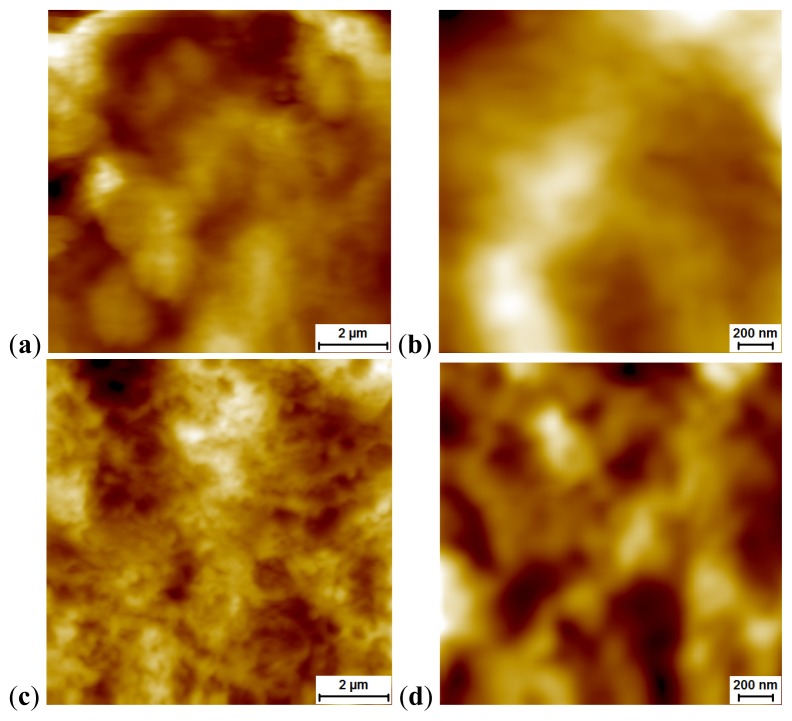

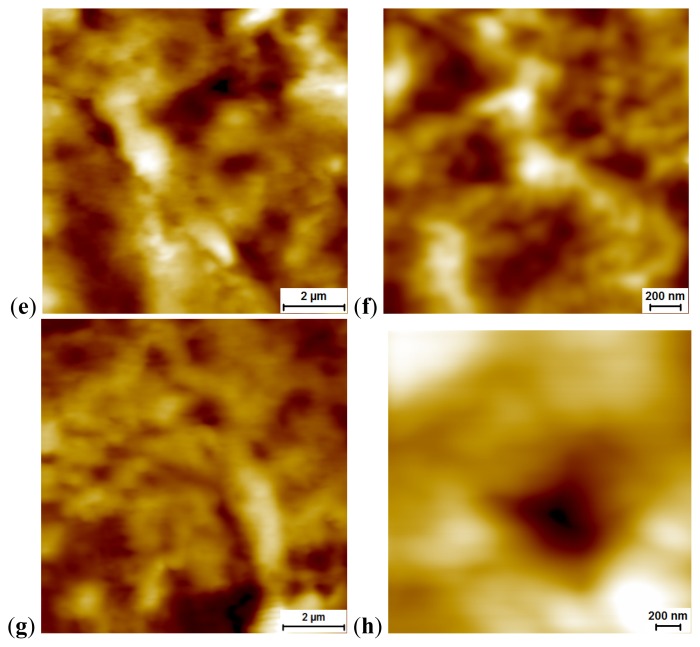
Typical cell surface topography imaged by semi-contact atomic force microscopy (AFM), for various groups of samples: (**a**) and (**b**) controls; (**c**) and (**d**) after instillation of 50 nm gold; (**e**) and (**f**) 49 nm silver; (**g**) and (**h**) 1 μm silver. Scan sizes: (**a**), (**c**), (**e**), (**g**) 10 × 10 μm^2^; (**b**), (**d**), (**f**), (**h**) 2 × 2 μm^2^.

**Figure 2:**
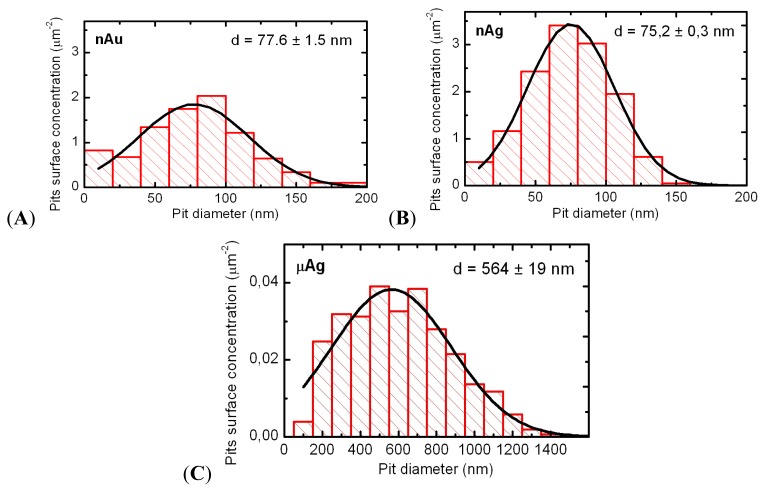
Histograms showing the distribution of pit densities by lateral dimension calculated for scans of small area (2 × 2 μm^2^) for cells interacting with nanogold (**A**); nanosilver (**B**); or microsilver (**C**).

The results of the present study, in general, corroborate those obtained earlier. To demonstrate this, we provide typical microphotographs of “pits” (Figure 1) and their distributions by diameter (Figure 2) for 2 μm × 2 μm scans focused at sites with the highest density of pits visible under whole-cell sc-AFM. Atomic force microscopy of such small scans makes it possible to enhance resolution and to visualize the relief’s details of up to 20 nm and, thus, more accurately study the distribution of the density of pits by diameter, including the smallest ones.

The average diameter of a pit at exposure to NG (77.6 ± 1.5 nm) is somewhat higher than at exposure to NS (75.2 ± 0.3 nm), which correlates with a small difference between the diameters of corresponding NPs (50 and 49 nm, respectively). The earlier observed [5] fact that the average pit size is slightly bigger than the average size of NPs is easily explainable, as the former is not a sharp “hole” made in the cellular plasma membrane by the passage of a particle through it but rather an image of an originally sloped micro-depression (invagination). On the other hand, the gradual deepening and closing of the mouth of this micro-pouch, before its complete sequestration from the cell surface, accounts for the fact that, at a given moment, a considerable number of pits have a diameter which is already less than that of the engulfed NP.

Similar to the experiments with magnetite particles, exposure to micrometric NS particles produced pits of a considerably larger diameter (564 ± 19 nm on average) than exposure to nanometric ones. It should be noted, however, that, in this case, the average pit diameter is about two times smaller than the average diameter of microparticles (1100 nm).

The average density of “pits” per unit area was found to be equal to 9.07 μm^2^ for NG and 13.14 μm^2^ for NS, *i.e.*, again 1.45 times higher for the more cytotoxic nanosilver.

#### 2.1.3. Transmission Electron Microscopy (TEM) Data

**Figure 3:**
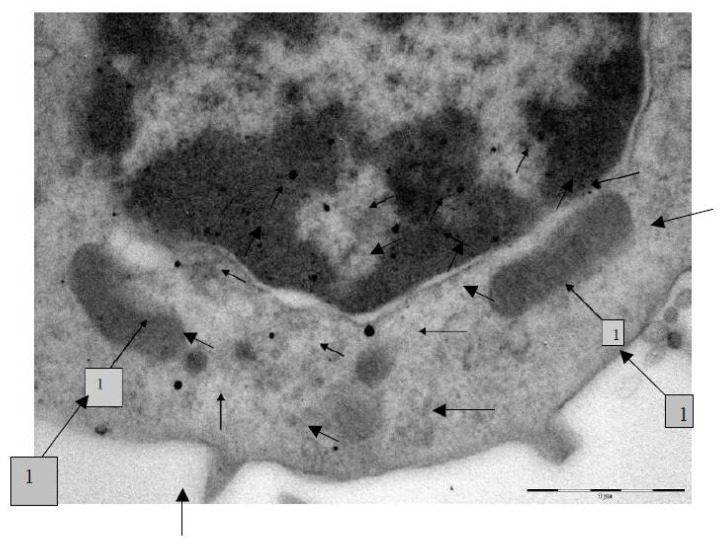
Gold nanoparticles are uniformly distributed throughout the cytoplasm and nucleus (non-numbered arrows) of an alveolar macrophage. The two-contour organization of the nucleus membrane is intact throughout. There is a mitochondrion visible (arrow 1) which is not interacting with nanoparticles but, nevertheless, is intact only partly. TEM, magnification 22,000×.

Nanogold. In each cell examined, we discovered from 15 to 20 gold NPs. This is illustrated by the AM microphotograph (Figure 3). Penetration through the plasma membrane occurs along with the formation of a phagosome separated by a thin membrane. In the cytoplasm, NPs are mainly found within phagosomes, typically as singlets and not contacting with any organelles. No aggregates of NPs were discovered within any of the examined cells. As few as 15 particles were seen inside the mitochondria in all of the 72 microphotographs of AMs and NLs, the particles being localized on the cristae or on the inner surface of the mitochondrial membranes. Such mitochondria demonstrate marked destruction of cristae, homogenization of the mitochondrial matrix, the two-contour membranes only partly remaining intact. Mitochondria demonstrate signs of destruction even when they are not in direct contact with particles present in the cytoplasm (presumably, an effect of Au-ions released by NG particles) though less marked than where NPs themselves have penetrated into the organelle. Lysosomes are observed in the cytoplasm in about the same quantity as in the control preparations.

Gold NPs are also seen in the nuclei of all cells examined. In some cases, particles penetrate into the nucleus without any marked change in the nuclear membrane; in other cases, particle penetration is accompanied with the blurring of the nuclear membrane and disturbance of its two-contour image. Similar changes in the nuclear membrane are observed where there are many particles near the nuclear membrane. Almost no changes are revealed in the chromatin around NPs in the nucleus, with only single instances of its thinning.

All the above is equally characteristic of AM and NL.

Nanosilver. Silver nanoparticles are not found in every cell—where they do occur, their quantities range from solitary NPs or moderate numbers, to considerable amounts. Unlike NG, NS is not found near the plasma membrane, either inside or outside the cells. They are mainly localized in deep regions of the cytoplasm, probably having had migrated there over the time interval from the moment of their *in vivo* contact with the cell to the moment of its drying. Comparison with the above-described picture of intracellular NG particle distribution suggests that NS particles migrate faster. Nevertheless, in one of the TEM pictures, we did discover a two-particle aggregate that had just penetrated through the plasma membrane with the formation of a phagosome. Phagosomes located deeper in the cell contain both single particles and their aggregates. However, it is impossible to judge whether the latter had penetrated into the cells in preformed state or had formed inside merged phagosomes. (The second mechanism seemed more likely for 10 nm particles of iron oxide, judging by the pattern of their distribution inside AM [4,5]).

**Figure 4:**
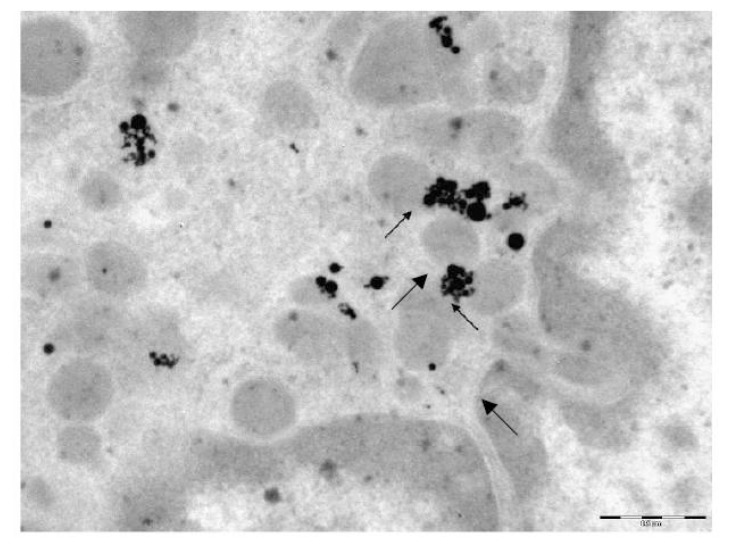
An alveolar macrophage. Penetration of silver nanoparticles from aggregates in the cytoplasm into mitchondria. No silver nanoparticles are discovered in the nucleus. TEM, magnification 28,000×.

**Figure 5:**
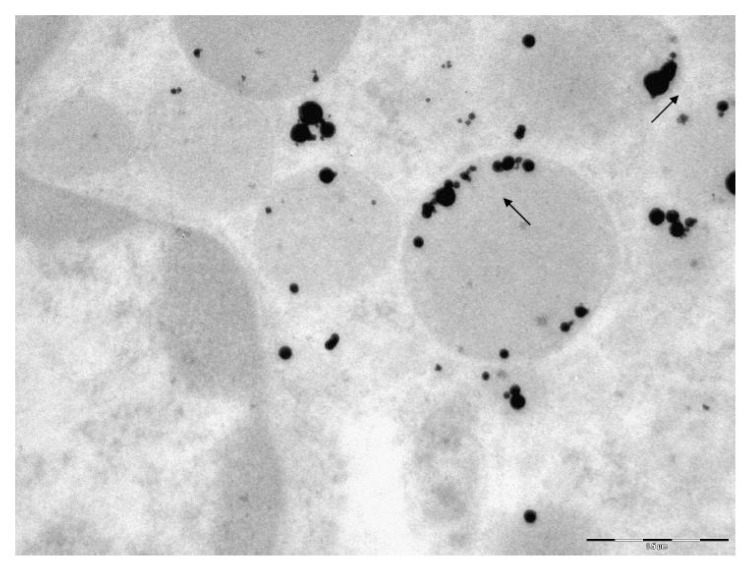
An alveolar macrophage. Silver nanoparticles are visible as singlets and aggregates on the inner surface of the mitochondrial membrane. Observable are cristae destruction, and mitochondrial matrix homogenization, with the mitochondrial membranes being only partly intact. No silver NPs are found in the nucleus. TEM, magnification 36,000×.

Particle aggregates are most often localized inside mitochondria either on the cristae or on the inner surfaces of their membranes (Figure 4 and Figure 5). In some mitochondria, these aggregates are so large that they occupy almost the entire organelle, but sometimes single NPs are also found inside the mitochondria. Both mitochondria that closely interact with particles and those that are free from direct contact with them demonstrate signs of destruction: only part of the cristae is intact, or just their fragments are visible; homogenization of the mitochondrial matrix is observed; the two-contour membrane is fragmented or is not found at all. Lysosomes are observable in the cytoplasm in approximately the same quantity as in the control preparations.

No silver NPs have been revealed inside the nucleus in any of the cells examined. In just one case, there is a large aggregate of particles in a phagosome near the nucleus, and in another TEM image a single NP is observed getting through the nuclear membrane without breaking the latter. Singlets are also observable near the nucleus without direct contact with it, and, in such cases, changes in the nuclear membrane are non-uniform. It may be blurred, and its two-contour organization damaged near a large singlet, but, at the same time, the nuclear membrane near a small aggregate consisting of medium-sized particles may be distinct, with a clearly visible two-contour organization.

Similar to NG, no difference between AM and NL as concerns the localization of internalized NS particles has been revealed.

Thus, the principal distinctions in the electron-microscopy images of pulmonary phagocytes exposed to NG and NS particles may be summarized as follows:

- NG particles are observed mostly as singlets and are fairly uniformly distributed, while NS particles tend to form aggregates
- Unlike NG particles, NS ones are virtually absent in the cell nuclei.
- NS particles tend to show greater tropism towards mitochondria than NG ones, accumulating in greater quantities within them and causing more marked destruction of the membranes and cristae.

It is also interesting to compare electron microscopy images at exposure to NG and NS with those obtained by us earlier in a methodologically similar study of the effects of nano-Fe_3_O_4_ [4,5]. It may be noted that changes caused by that nano-substance are, in certain aspects (propensity to the formation of particle aggregates and accumulation in mitochondria with marked damage of the latter, and absence of any visible nanoparticles within the nucleus) were closer to changes caused by nanosilver than to those caused by nanogold. At the same time, exposure to nano-Fe_3_O_4_ produced clearly visible invaginations in the cell membrane with the formation of minute phagosomes, a great number of which (as well as of randomly distributed free nanoparticles) were located at the cell periphery, while in the case of contact between NPs aggregates and the nuclear membrane, the latter showed distinct destruction. Notable was also the absence of lysosomes, an effect we do not observe at exposure to both NS and NG. However, it is hard to tell whether these distinctions are attributable to unequal properties of the compared metals or to the fact that the earlier TEM-investigated nano-Fe_3_O_4_ particles were much finer (10 nm) in comparison with the NS and NG particles investigated in the present study. Most likely, both the intracellular distribution of metal nanoparticles within phagocytizing cells and ultrastructural changes in the latter are not universal, but are dependent on both the chemical nature of the metal and particle size.

### 2.2. Effects of Repeated Intraperitoneal Injection

For studying the comparative subchronic toxicity of NPs differing either in diameters [2,3] or in chemical nature (this study) we decided to evade the issue of the intricate dependencies of their systemic effects on different physiological and other mechanisms underlying the kinetics of deposited nanoparticles’ elimination from, or retention in, the lung tissue. These dependencies would have substantially complicated the interpretation of comparative toxicity at organism level. Meanwhile, our goal was just to reveal those differences in comparative systemic toxicity of NS and NG that are associated with mechanisms of toxicokinetics specific for NPs (such as active dissolution and resorption from the primary depot; transfer of not only dissolved material but also NPs as such to remote organs by blood; secondary retention of migrating NPs in these organs, chiefly those rich in RES cells; their possible dissolution in these secondary depots). Therefore, it was desirable to have an experimental model in which the mass of the material in the primary depot would be strictly set and equal for substances under comparison. From this standpoint, a sufficiently adequate experimental model for subchronic toxic exposure to compared NPs seems to be repeated intraperitoneal injections of their suspensions in not lethal doses during a period which is long enough relative to the rat’s lifespan.

Certainly, it should be borne in mind that barriers through which particles penetrate into the blood from the lungs and from the peritoneal cavity are different anatomically and functionally, and these differences can be reflected on NPs’ toxicokinetics. One may well assume, however, that this fact does not create a major bias for comparative estimation of the capacity of particles of different size or chemical nature for such penetration and migration to remote organs. It should be noted that intraperitoneal administration was used for studying the resorptive toxicity and bio-accumulation of some NPs not only by us in relation to nano-Fe_3_O_4_ [2,3], but also by other authors investigating effects of other nanometals, in particular, of nanogold [60,61].

#### 2.2.1. Indices of Systemic Toxicity

As can be seen from Table 2, just a few of the 36 functional indices to the condition of the organism at exposure to NG or NS that we used differ statistically in a significant manner from corresponding indices for the control group. In particular, exposure to NG is observed to decrease the hemoglobin content and RBC count in the blood, to increase the percentage of monocytes in the hemogram, to reduce the activity of succinate dehydrogenase (SDH) in blood lymphocytes; and to decrease malondialdehyde (MDA) urine excretion, and kidney mass. Exposure to NS significantly reduces the activity of SDH and the level of ceruloplasmin (the principal copper-containing protein of the blood) [62]. The latter shift, observable (though statistically not significant enough) at exposure to NG as well, is, probably, the consequence of the hepatotoxicity of these nanometals (as the synthesis of serum ceruloplasmin is performed by hepatocytes) or results from the mutation caused by them in the ceruloplasmin gene (*CP*) that controls this synthesis. Note, however, that in the earlier experiment of similar design with nano-iron oxide, administered in a much higher dose, we observed a number of indices to obvious damage of the liver, but no changes in the ceruloplasmin concentration in the blood were present [2,3] Thus, its decrease in response to exposure to NG and especially to NS is relatively specific, reflecting, possibly, competitive relations between silver and gold on the one hand, and copper on the other.

Given such insignificant shifts or the absence of any shift, differences in the toxicities of compared agents are never sufficiently manifest. Indeed, difference in the effect of NS and NG proves to be statistically significant just for two indices only. One of them is a reduction in ceruloplasmin content, which is a lot more pronounced in response to NS than to NG. The second is enhanced lipid peroxidation, estimated by an increase in the level of MDA excretion. Although at exposure to NS this increase compared with the control level is statistically insignificant, it is significantly higher than in the NG group.

A decrease in the RBC count in comparison with the controls, significant for both nano-metals, is also more marked in response to NS, although the difference from the corresponding effect of NG is not sufficiently significant statistically. At the same time, an increase in the percentage of monocytes in response to NS is less marked than to NG, and the decrease in SDH activity is almost identical (even slightly less marked in response to NS).

Thus, the dose of both nano-metals used in this experiment proved to be close to LOAEL (Lowest Observed Adverse Effect Level), with equivocal and poorly expressed differences between the resulting shifts in various systemic toxicity indices. At the same time, there might be an objective reason why NG and NS, whose acute toxicity at cellular level has been found so obviously unequal (Table 1), feature almost equal subchronic toxicity at systemic level. Specifically, some target organs accumulate more gold than silver, and just this difference could partially offset oppositely directed difference in toxicity for the cells of the same organs.

**Figure 6:**
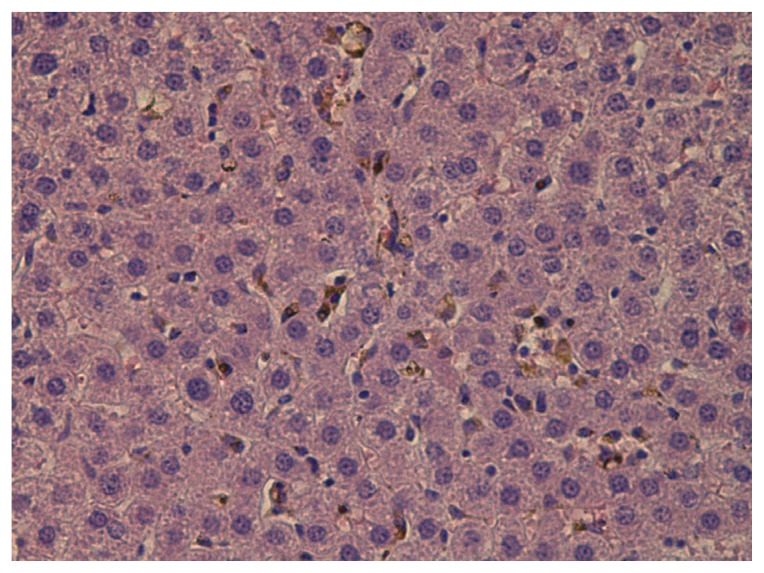
The liver of a rat exposed to nanosilver. One can see a large number of macrophages with particles in the dilated sinusoids of a hepatic lobule’s central part. Hematoxylin and eosin staining, magnification 200×.

Nevertheless, histological examination of the liver, kidneys and spleen has revealed some clear pathological changes. Thus, microscopy of liver sections of rats from all experimental groups (but not from the control group) shows brown and gold-brown colored granules in the cytoplasm of the macrophages (Kupffer cells), which are likely to be aggregates of administered nanoparticles (Figure 6). (It is known that “Kupfer cells are central in the removal of nanoparticles from the organism” [60].) Hepatocytes demonstrate moderately expressed dystrophic changes. Mononuclear cells and, occasionally, polymorphonuclear leukocytes are notable in the sinusoids, though in small quantities. Portal stroma, even where particles have been deposited in it, is not changed.

No appreciable differences in the histological picture of the liver have been found in response to the administration of NG and NS.

Judging by the morphometric indices shown in Table 3, both nanometals have caused just a small and statistically insignificant increase in the number of akaryotic hepatocytes, which is an important index of hepatotoxicity. However, the number of binucleated hepatocytes, an increase in which points to reparative enhancement of mitotic activity, is somewhat higher than in the control group, this shift being statistically more significant for NG. It may be noted that, earlier, we found that subchronic exposure to higher doses of iron oxide NPs [2,3] caused a sharp increase in the number of akaryotic hepatocytes and a decrease in the number of binucleate cells, and the histological picture demonstrated marked damage to the structure of the hepatic lobules. In the current experiment, however, the only essential morphometric index of changes in the liver marks a 1.5 fold increase in the number of Kupffer cells. Although this change is almost identical in response to NS and NG, the semi-quantitative index of cell particle burden is significantly, 2.6 times higher in the first case. This difference may, presumably, be explained by the fact that the impact of more cytotoxic NS caused more extensive destruction of macrophages (Kupffer cells), as we had demonstrated earlier [56] that macrophage breakdown products enhance the phagocytic activity of viable macrophages *in vitro*.

The spleen of rats exposed to both nanometals display few small aggregates of NPs in the center of the lymphoid follicles. A morphometric index of the functional status of the spleen under various types of stress might be the ratio of areas occupied on a section by the white and red pulp (e.g., [62]). The share of the white pulp has been found to be equally lower in response to both nanometals (Table 3).

**Figure 7:**
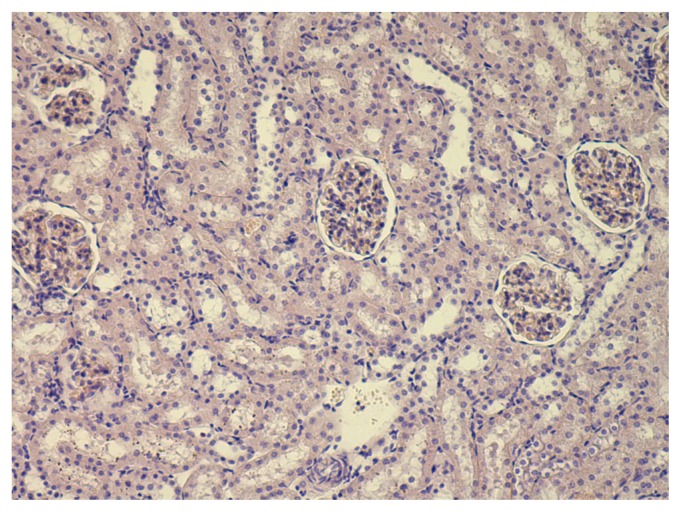
The kidney of a rat exposed to nanosilver. One can see glomeruli with non-uniform proliferation of mesangiocytes. The globular basal membranes are pronounced, with brown coloring. Small granular deposits are seen in the mouths of the tubules, in the cytoplasm of tubular epithelium cell, and in the peritubular stroma. Moderate dystrophic changes in the tubular epithelium. Hematoxylin and eosin staining, magnification 100×.

**Figure 8:**
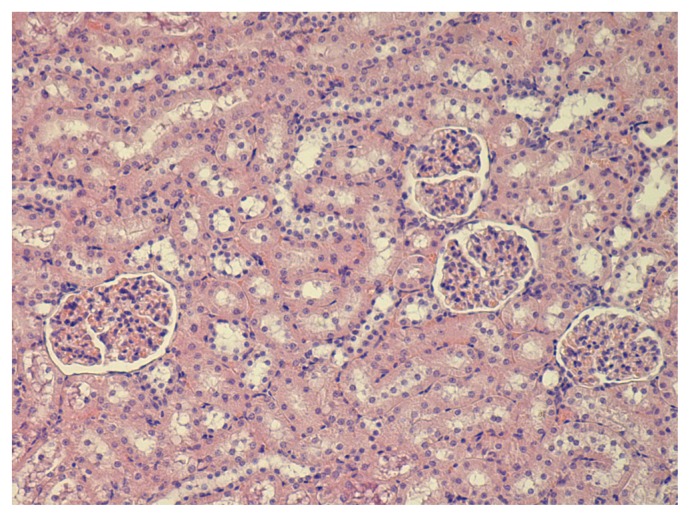
The kidney of a control rat. Hematoxylin and eosin staining, magnification 100×.

The kidneys of the experimental animals demonstrate moderate thickening and a clearly enhanced contour of the glomerular basal membranes (Figure 7 and Figure 8 for comparison with control kidney). This picture resembles rather closely the one that could be seen when tissue sections are impregnated with silver, and so we deal, most likely, with some kind of vital silvering of the membrane structures associated with the glomerular filtration of Ag-ions released from NS particles both in the suspensions and, obviously, *in vivo*. It is hard to tell whether this impregnation with silver is pathogenetically significant. Measurement of the Malpighian corpuscle dimensions, including the external diameter (*i.e.*, across the parietal layer of the Bowman’s capsule) or the diameter of the glomerulus (*i.e*., across the visceral layer of the capsule) has not revealed any changes in response to NS whereas both dimensions are statistically increased in a significant manner at exposure to NG. To illustrate this, here are the values of the diameter as measured (in μm) across the parietal layer of the capsule: 4.18 ± 0.07 for the control group, 4.67 ± 0.07 * for the NG group, 4.27 ± 0.07 for the NS group (the sign * points to the index which is statistically and significantly different from the control group, *p* < 0.05).

Single fine particles of brown or gold-brown color are occasionally observed in the mesangial areas of the glomeruli of animals that received any of the nanometals. Particles of various sizes are found in the tubulur epithelium and in their mouths.

It should be noted that the functional indices (Table 3) have not revealed any obvious impairment in the condition of the liver or kidneys. Contrary to popular belief, structural changes in these organs, even at cellular level, proved to be a little more sensitive index of the toxic action.

#### 2.2.2. Genotoxicity

Judging by the results of the RAPD test presented in Table 4, both NG and NS are genotoxic, the genotoxicity of nanosilver being noticeably higher. The coefficient of fragmentation (Cfr), statistically significant in its elevation compared with its value for the control group in the case of NG is observed only in the bone marrow and kidney, while values elevated statistically insignificantly are also found in blood cells and spleen. At the same time for NS, significantly increased values of Cfr are obtained in all examined tissues except for the skeletal muscle. If we compare the groups of rats exposed to these nanometals between them, we can see that in all organs, except for kidneys, Cfr is higher in response to NS than to NG, this difference being statistically significant for the liver and spleen.

Comparing these facts with the literary data succinctly summarized in the Introduction, we can state that the genotoxicity of both nanometals has been, for the first time, convincingly shown by us for subchronic exposure of a whole organism, and for the DNA of various tissues rather than on cultured cells. The fact that the genotoxic effect is present in different tissues to a different degree may, most likely, be explained by: (a) unequal intensity of mitoses in them (because the stripping of the nuclear membrane in the course of mitosis, starting from the prometaphase and including the meta- and anaphase, renders the genomic DNA more accessible to a contact with a damaging factor); (b) unequal probability of retention and accumulation in these tissues of NPs that penetrate from the primary depot in the abdominal cavity into the lymph or directly into the blood. The probability of secondary long-term retention of migrating NPs in an organ is determined, in turn, by (a) the quantitative characteristics of its perfusion and permeability of the histohematic barriers and (b) the abundance of cells of the Reticulo-Endothelial System (RES) capable of entrapping these NPs. The fact that skeletal muscles have proven to be the least susceptible to the genotoxic action of NPs *in vivo* is, most likely, explained by the shortage in them of both mitoses and the RES cells.

We have measured the actual concentrations of both metals only in three tissues: liver, spleen and kidneys (Table 5). In the control group, the gold and silver content of these tissues was close to zero. In the NS and NG groups, corresponding metals were found in the liver and spleen in a concentration of the same order of magnitude, although there was a little more gold in them than silver (for the liver this difference is statistically significant). On the contrary, the kidneys contained 30 times less gold compared with the concentration of silver. One may assume that this organ, poor in phagocytizing RES cells, accumulates not so many NPs as the metal that filtrates in ionic form through the renal globules from the blood. The less soluble NG is likely to release ions into the blood to a considerably lesser degree than NS. (According to the data of other researchers summarized in [26], however, after a single intravenous injection of a sufficiently high dose of 50 nm NG particles, gold is detected in the kidneys, but in a much smaller concentration than in the liver and spleen.)

On the other hand, the same lower solubility of NG particles in comparison with NS particles may be the most likely cause of the greater bio-persistence of the former in the RES-abundant target organs’ secondary depots of the NPs as such. To confirm this hypothesis we have experimentally investigated the process of reduction (from the initial value of 0.025 mg/mL) in the concentration of NPs in normal saline diluted with water in a ratio of 3:1, using 400 nm wavelength light absorption. Measurements were carried out during one day. Before each measurement, ultrasonic dispersion was performed for 10 min. We observed exponential reduction in light absorption with time constants of about 2.3 h for gold and 3 h for silver. After 24 h of exposure of the nano-suspensions, light absorption went down to 58% of the initial value for NG and to only 11% for NS. This testifies to more complete disappearance of NS particles due to their dissolution.

That the concentration of metal retained mainly in the form of NPs by RES cells is higher in the spleen than in the liver has already been observed by us in a similar experiment with subchronic exposure to iron oxide NPs [2,3]. Meanwhile, as can be seen from Table 3, both a relatively low genotoxic effect of NG, and a markedly higher effect of NS, is almost identical for these organs. It is likely that lower accumulation of NS in the liver is “outweighed” by the higher regenerative activity of this organ.

It is important from the theoretical point of view that, given comparable NP sizes and exposure conditions, NS proves to be substantially more bioactive in terms of genotoxicity for all tissues (as well as in terms of cytotoxicity for pulmonary phagocytes) than NG. Meanwhile, the electron microscopy results considered above show that NG particles prove to be much more capable of penetrating into the nucleus of phagocytizing cells *in vivo* than NS particles (possibly, due to a higher propensity of the latter to intracellular aggregation). This apparent paradox may be considered as indirect evidence in favor of the widespread belief that the genotoxicity of both nanometals is associated not so much with direct interaction between NPs and nuclear DNA as with damage to this DNA by reactive oxygen species penetrating into the nucleus from the cytoplasm, mainly from mitochondria generating these ROS under the effect of NPs (oxidative stress). Indeed, as it was shown above, electronic microscopy had revealed that both the accumulation of NPs in mitochondria and damage to mitochondrial membranes and cristae at exposure to NS are much more considerable than at exposure to NG. Association between the accumulation of ultrafine particles of atmospheric dust in mitochondria and displays of oxidative stress has been observed by other researchers in experiments on cells *in vitro* [63].

On the other hand, from the point of view of regulatory toxicology and of NP-induced health risk assessment, it is crucially important that for both nanometals studied by us the genotoxicity *in vivo* is clearly manifest at a level of exposure so low that their systemic toxic action on the organism is only slightly expressed. Apparently, it is just genotoxicity (and thus, probably, carcinogenicity) rather than systemic toxicity that should be considered as a limiting risk for certain nanomaterials. Judging by our results, both NG and, especially, NS are such nanomaterials.

Therefore, the search for protectors against not only the systemic toxicity of such NPs, but also their genotoxic effects, acquires special importance, and, thus, the feasibility of such biological protection as demonstrated in the next subsection seems to be not only a new, but also a very important discovery.

#### 2.2.3. Effects of the Tested Bioprotective Complex (BPC)

As can be seen from the Table 4, the lower average values of Cfr in rats exposed to NS and receiving bioprotectors (the “NS + BPC” group) compared with rats receiving NS only, are found in all the tissues studied, this difference being statistically significant for liver, bone marrow, spleen and kidney. The anti-genotoxic action mechanisms of the bioprotectors selected for testing are complex and, apparently, mutually potentiating. We believe that the following may be of great importance: (a) the different (in terms of molecular mechanisms) antioxidant effects inherent, to a certain extent, to a number of bioprotectors in the complex used (antioxidant synergy); (b) the membrane stabilizing action of glutamate because it can prevent damage to mitochondria and, thus, oxidative stress; (c) the toxicological antagonism between some trace elements (selenium, copper, calcium) and silver, the mechanisms of which are diverse and are not yet quite clear.

As for the functional indices of nanosilver’s systemic toxicity, being weakly expressed even without biological protection of the organism as shown above (Table 2), they have not dramatically changed with bioprotection (Table 6). Nevertheless, judging from the shifts in the indices which are statistically significantly different in comparison with controls at exposure to NS without protection, their attenuation at exposure to the same NS plus BPC is obvious. Thus, the RBC count, which is considerably decreased in the NS group, does not differ from the control value at exposure to the same NS plus BPC. It is also important that the hemoglobin content (whereby the statistical decrease by the NS without BPC was not significant, yet nevertheless obvious) and the percentage of reticulocytes (elevated in the NS group in comparison with the controls, though statistically not sufficiently significant) in the group given NS against the background of BPC administration did not differ at all from respective control values. MDA excretion was also normal, but not that of ceruloplasmin. The concentration of delta-aminolevulinic acid in the urine decreases significantly (compared with the group receiving NS without BPC). However, this shift is unlikely to be significant toxicologically, as the action of NS itself did not cause any increase in this index, and BPC itself did not cause any decrease in it.

The toxicological interpretation is much more unequivocal for the intergroup differences in terms of the lymphocyte’s SDH activity, a very sensitive integral index for assessing the redox metabolism on organism level. A significant decrease in this index was observed in numerous experiments involving exposure to various toxic factors, it becoming either partially or fully normal if relevant BPC were administered along with toxic dosing (e.g., [64–66]). An essential decrease in this index was also observed at exposure to iron oxide NPs of both 10 nm and 50 nm in diameter, but not to micrometric particles of the same substance [2,3].

As for the current experiment, this index is also significantly reduced compared with the control value when rats are exposed to just NS, whereas it is even slightly elevated at exposure to the same NS plus BPC or to the BPC only.

It is important to emphasize that there is no single index for which statistically significant differences would be revealed between the groups receiving BPC only and controls. Thus, the absolute requirement that any bioprotective complex developed and tested with an eye to its preventive application in practice be harmless has been met.

Histological examination of the liver, spleen, and kidneys has also demonstrated a beneficial effect of the bioprotective complex. We should refer the reader back to Table 3 and note that, compared with the NS group, the NS plus BPC group features a statistically significant reduction in the number of Kupffer cells, their particle burden and the number of akaryotic hepatocytes (which proved to be even significantly lower than in the control group), while the reparative index of the number of binucleated hepatocytes, on the contrary, proved to be statistically significantly, 1.5 times, higher.

As can be seen from the same Table, in the spleen the BPC prevented a reduction in the white to red pulp ratio caused by NS, which in the NS + BPC group does not differ from the control value.

In the kidneys, the aforementioned (Section 2.2.1) tubular changes were also noticeably reduced under the effect of the BPC. Along with this, the “silvering” of the glomerular basal membranes (revealed in rats exposed to NS only) was not observed in the group exposed to NS plus BPC. It is hardly probable that the action of the bioprotectors could have weakened the release of Ag-ions, which we proposed above as an explanation for this phenomenon. However, it is likely that the action of BPC caused a metabolically conditioned shift in pH or any other physicochemical changes in the renal tissue which prevented the retransformation of Ag-ions into “nuclei” of elemental silver. Just such metallic nuclei may be supposed to get deposited on the membranes, keeping in mind that this transformation is, obviously, a key mechanism of the different histochemical techniques of silver impregnation [67]. A decrease in the kidneys’ silver content in the NS + BPC group as compared with the NS one is not statistically significant and so cannot corroborate this hypothesis. Nonetheless, it does not contradict it.

We believe, however, that all the other beneficial effects described above of the BPC tested are associated with different toxicodynamic mechanisms, rather than with an influence on the NS toxicokinetics (distribution, excretion, retention in the organism) because, as follows from Table 5, BPC has not had any significant effect on the silver content of the tissues.

## 3. Experimental Section

All experiments were carried out on outbred white female rats from our own breeding colony. There were 8–14 animals in different exposed and control groups, with the initial body weight of 150 to 220 g. Rats were housed in conventional conditions, breathed unfiltered air, and were fed the standard balanced food. The experiments were planned and implemented in accordance with the “International guiding principles for biomedical research involving animals” developed by the Council for International Organizations of Medical Sciences (1985).

For our study, we have prepared stable suspensions of silver and gold nanoparticles by the method of laser ablation in liquid, including the following main phases: (a) laser ablation of the metal target in water, and, where necessary, (b aser fragmentation for preventing particle aggregation, and (c) concentration of the suspension. In our case, concentration was necessary to enable the administration of effective NP doses to rats in minimal volumes of water.

A plate of silver or gold with a metal content of 99.99% was placed on the bottom of a dish with deionized water. Metal ablation was performed using Fmark-20RL laser material processing system (by Laser Technology Center, Zelenograd, Russia) based on ytterbium-doped pulsed fiber laser (pulse length 100 ns, repetition rate 21 kHz, wavelength 1064 nm). The energy density was 80 J/cm^2^. The target was Zirradiated in scanning mode with a rate of the laser ray 270 mm/s.

**Figure 9:**
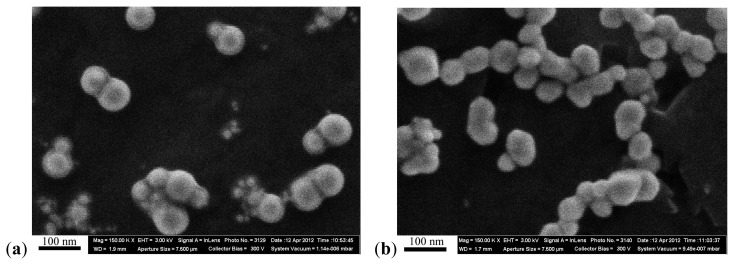
Images of nanoparticles in suspension obtained by scanning electron microscopy at 150,000× (**a**-nanogold; **b**-nanosilver).

**Figure 10:**
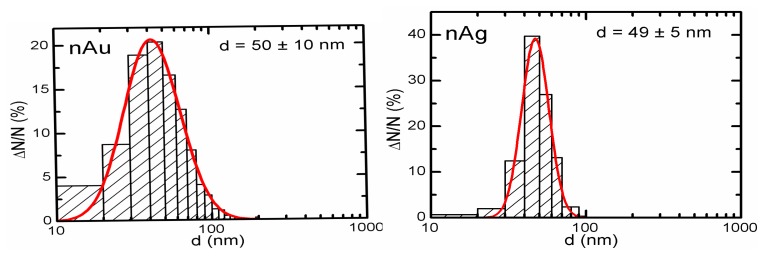
Nanoparticle size distribution function: the results of statistical processing of 800 images of gold NPs (left) and 650 images of silver NPs (right) obtained by scanning electron microscopy.

The concentration of the primary suspensions obtained by ablation was 0.10 mg/mL for gold, and 0.12 mg/mL for silver. Fragmentation proved necessary for preventing the aggregation and for enhancing the stability of the nanogold suspension, involving after-treatment with a laser ray focused into the bulk of the suspension. An increase in concentration to 0.5 mg/mL was achieved by drying the suspensions which was not accompanied by nanoparticle aggregation. Particle images were obtained after concentration by scanning electron microscope (SEM), AURIGA CrossBeam Workstation (Carl Zeiss, Oberkochen, Germany), which enabled us to identify their spherical form (Figure 9). Average NP diameters (± s.d.) obtained through statistical processing of hundreds of SEM images were: 50 ± 10 nm for NG and 49 ± 5 nm for NS, the distribution being symmetrical (Figure 10). Nanogold average particle size was also measured by the dynamic light scattering method by the Zetasizer Nano ZS analyzer (Malvern Instruments, Worcestershire, UK), and provided a sufficiently close result, 58 ± 19 nm.

No essential changes took place 30 days after the preparation of the suspensions in either zeta potential or the form and position of the plasmon resonance peak, providing evidence of their high stability.

The suspension of silver microparticles with an average diameter of 1.1 μm was obtained by burning ash-free filters impregnated with silver nitrate, with subsequent ultrasonic dispersion in deionized water. All the above described stages and regimes of suspension preparation had been preliminarily optimized in a series of experiments.

Both NS and NG suspensions were administered to rats:

- either once intratracheally (i.t.), at a dose of 0.5 mg in 1 mL of the freshly prepared suspensions of NG or NS (in parallel with the same dose of silver microparticles);
- or, intraperitoneally, three times a week (up to 20 injections) at a dose of 10 mg/kg in the corresponding volume of the suspension containing 0.5 mg of NS or NG per mL (This concentration was highest for stable nano-suspensions that we were able to obtain with the technique described above, while the biggest tolerable volume of an i.p. injection to a rat was found to be 4 mL. Just these two factors determined the actual dosage of NS and NG, even if we wanted it to be somewhat higher for being more certain with regard to the toxic effects of these metals. However a preliminary short-term (2 weeks) pilot experiment with the presumably least toxic NG demonstrated that even the actually attainable low dosage was not without some adverse effects).

Animals in respective control groups were administered sterile deionized water (from the batch used for preparing suspensions) by the respective route. In the subchronic experiment, an additional group of rats studied in parallel was being injected with the same dosage of NS, but against the background of administration of a bioprotective complex (BPC) described below, and still another group was given the same BPC plus i.p. injections of water.

In the acute intratracheal test, along with optical microscopy of cells sedimented by centrifuging the BALF, obtained as described below, we examined the topography of the BALF cells surface. To this end, we used semi-contact atomic force microscopy (sc-AFM) reputed as a unique technique allowing one to obtain 3D visualizations of the surface topography of biological objects with a nanometric spatial resolution. A 3 μL aliquot of the BALF sediment was precipitated on a fresh cleavage of mica. After 60 s, excessive suspension was removed with a paper filter, and the sample was dried by blowing with clean, dry air or nitrogen for 30 s. It should be noted that the drying of the BALF on a mica surface results in the formation of salt microcrystals, which were removed by washing the sample twice. For washing, the sample was kept for 60 s on the surface of a drop of deionized water (with the working side down). The liquid was then removed with the help of a paper filter. After repeating the washing, the sample was dried by blowing with clean, dry air or nitrogen for 30 s. Investigation of the cell surface morphology was performed by sc-AFM with the help of an NTEGRA Therma scanning probe platform by NT-MDT (Zelenograd, Russia) using semi-contact atomic force microscopy mode with NSG01 probes by NT-MDT (Zelenograd, Russia). The height of the probes was about 15 μm, and the tip curvature radius was less than 10 nm. For statistical processing and analysis of measurement results, we used specialized software, SPIP (Image Metrology, Horsholm, Denmark) and SIAMS Photolab (Ekaterinburg, Russia). The procedures developed made it possible to reveal the pits in the images of cell surfaces and to measure the diameter and depth of each pit. The results of image analysis were used for plotting the histograms of pit dimensions for cells of all groups of rats.

The transmission electron microscopy (TEM) was used to study the localization of different NPs within the BALF phagocytes and to visualize damage to the cells at ultra structural level that may be attributed to the cytotoxic effect of NPs.

Bronchoalveolar lavage was carried out 24 h after the i.t. instillation of suspensions or reference water. A cannula connected to a Lüer’s syringe containing 10 mL of normal saline was inserted into the surgically prepared trachea of a rat under hexenal anesthesia. The fluid entered the lungs slowly under the gravity of the piston, with the animal and syringe positioned vertically. Then the rat and the syringe were turned 180°, and the fluid flowed back into the syringe. The extracted BALF was poured into siliconized refrigerated tubes. An aliquot sample of the BALF was drawn into a WBC count pipette together with 3% acetic acid and methylene blue. Cell count was performed in a standard hemocytometer (the so-called Goryayev’s Chamber).

For cytological examination and semi-contact atomic force microscopy (sc-AFM), the BALF was centrifuged for 4 min at 1000 rpm, before the fluid was decanted, and the sediment was used for preparing (a) sc-AFM samples as described above, and (b) smears on 2 microscope slides. After air drying, the smears were fixed with methyl alcohol and stained with azure eosin. The differential count (under optical microscope with immersion at a magnification of 1000×) for determining the percentage of alveolar macrophages (AM), neutrophil leukocytes (NL) and other cells was conducted up to a total number of 100 counted cells. Allowing for the total number of cells in the BALF, these percentages were recalculated in terms of absolute AM and NL counts.

For performing TEM, BALF was centrifuged for 30 min at 3000 rpm. The cell sediment was fixed in 2.5% solution of glutaraldehyde with subsequent additional fixing in 1% solution of osmium tetroxide for 2 h. Then it was washed in 0.2 M phosphate buffer and passed through alcohols of increasing concentration and through acetone for dehydration. Then the sample was placed for 24 h in a mixture of araldite and acetone at a ratio of 1:1, following which it was polymerized in araldite at 37 °C for 1 day and at 50–60 °C for the following 2–3 days. Ultrathin sections were obtained on a Leica EM UC6 ultramicrotome (Wetzlar, Germany), contrasted with lead citrate and examined on a Morgagni 268 electron microscope (FEI Company, Eindhoven, The Netherlands).

In the subchronic intraperitoneal test, immediately after the subchronic exposure period, the following procedures were performed for all rats:

- weighing;
- estimation of the CNS ability to the temporal summation of sub-threshold impulses—a variant of withdrawal reflex and its facilitation by repeated electrical stimulations in intact, conscious rat [68];
- recording of the number of head-dips into holes of a hole-board, which is frequently used for studying behavioral effects of toxicants and drugs (e.g., [69,70]);
- collection of daily urine for analysis of its density, urine output, coproporhyrin, delta-aminolevulinic acid (δ-ALA), hydroxyproline, and creatinine contents;
- sampling of capillary blood from a notch on the tail for examining the hemogram, hemoglobin content, and for cytochemical determination of succinate dehydrogenase (SDH) activity in lymphocytes (by the reduction of nitrotetrazolium violet to formasan, the number of granules of which in a cell is counted under immersion microscopy).

Then the rats were killed by decapitation and blood was collected by exsanguination. Biochemical indices determined from serum included total protein, albumin, globulin, bilirubin, ceruloplasmin, malonyldialdehyde (MDA), alkaline phosphatase, alanine- and asparate-transaminases (ALT, AST), catalase, gamma glutamyl transferase. For four rats in each treatment and control group, liver, kidney, and spleen tissue sections were prepared for histological examination by staining with hematoxilin-eosine and with the Van Gieson’s stain.

### 3.1. Testing of Genotoxicity (the RAPD Test)

In total, we analyzed 96 samples, each sample in three replications. Muscular tissue was separated with a scalpel from the bone tissue. Samples of solid organs were minced with a scalpel. Then for the purpose of obtaining homogeneous mass, the samples were subjected four times to freezing in liquid nitrogen and to thawing in an ultrasonic bath (38 °C) in the Ca^2+^, Mg^2+^ free PBS solution (Sigma, St. Louis, MO, USA) with subsequent passing through needles of decreasing diameter. Bone marrow cells were washed out of the femur (after cutting off the epiphyseal and diaphyseal areas) with the same PBS solution with subsequent passing through needles of decreasing diameter. Nucleated peripheral blood cells were isolated from the whole blood of the laboratory animals by centrifuging in the Percoll gradient (Sigma, St. Louis, MO, USA).

To isolate DNA from the cells, we used a GenElute (Sigma, St. Louis, MO, USA) set of reagents in accordance with the manufacturer’s guidelines for use. In the samples obtained, we determined spectrophotometrically with Ultraspec 1100 pro (Biochrom Ltd, Cambridge, UK) the content of DNA and freezed and stored them at −84 °C in a kelvinator Sanyo (Moriguti, Japan) till the beginning of the analysis.

The Random Amplification of Polymorphic DNA (RAPD) test was performed as described earlier [65,66]. This technique allows one to define quantitatively the degree of DNA fragmentation as an estimate for the genotoxicity of harmful agents and for the protective effects of the complex of bioprotectors studied. The method is based on the fact that, unlike a fragmented DNA, which forms the so-called “comet tail” in the agarose gel in electrophoresis, a non-fragmented DNA has a very low degree of migration and virtually stays in the same place (“comet head”), and the degree of migration is directly related to the degree of DNA fragmentation. To characterize the degree of damage to DNA we used “coefficient of fragmentation”, *i.e.*, the ratio of total radio-activity of all “tail” fractions to the radioactivity of the “head”.

The gold or silver content of liver, spleen and kidneys was determined by atomic emission spectrometer with inductively coupled plasma iCAP-6500 Duo (Thermo Scientific, Billerica, MA, USA). Samples of freeze-dried homogenized tissue were subjected to acid digestion with the help of a MARS 5 microwave accelerated reaction system.

### 3.2. Choice of Bioprotectors

A review of the literature data on the mechanisms of toxic and genotoxic action of silver, combined with our experience in the testing of various bioprotectors for other intoxications (summarized in [49]), allowed us to choose, for estimating the possible protective action at subchronic intoxication with nanosilver, a complex that included the following biologically active substances:

- Glutamate as an effective cell membrane stabilizer through the intensification of ATP synthesis under exposure to the damaging action of various cytotoxic particles (e.g., [64–67,71]) and, at the same time, as one of the three precursors of glutathione, a powerful cell protector against free radicals.
- The other two precursors of glutathione: glycine and cysteine (the latter in a highly active and metabolically easily available form of *N*-acetylcysteine).
- Other components of the organism’s antioxidant system (vitamins A, E and C, and selenium).
- Trace elements that are physiological and toxicological antagonists of silver (selenium, copper, calcium) [16,72].
- Omega-3 polyunsaturated fatty acids, whose intracellular derivatives are eicosanoids that activate DNA replication and thus play an important part in its repair, which was demonstrated under exposure to various genotoxic agents (e.g., [64–67]).
- Pectine enterosorbent as an agent that prevents the reabsorption of the metal excreted into the intestines with bile.

Based on the experience of our earlier experiments ([64–67,71] and many others), we administered these bioprotectors in the following ways (see Table 7). We gave glutamate to rats as a 1.5% solution instead of drinking water *ad libitum*. “Eicosovitol” (by Farmavit Ltd., Tyumen, Russia), a fish oil preparation rich in PUFA pertaining mainly to the omega-3 group was administered through gavage at a dose of 1 mL per rat. The apple pectin enterosorbent (by Promavtomatika Ltd, Belgorod, Russia) was added to the rats’ fodder in a quantity corresponding to a dose *ca.* 1000 mg/kg body weight. Other commercial preparations of amino acids, vitamins and minerals available as tablets were crushed and added to another portion of the fodder in quantities corresponding to the recommended daily intake of these micronutrients by rats. (If such recommendations were known only for humans, a recalculation to the rat’s nutritional requirement was made based on the species’ standard metabolisms ratio.)

Taking into account that the standard balanced food presumably meets the normal nutritional requirements of a rat, we assumed that additional intake of the above-listed bioactive substances would meet the increased needs connected with molecular mechanisms of silver toxicity. Nevertheless, it had to be checked whether or not such “overloading” with them would engender any unfavorable effects. For this reason, in our subchronic experiment, one group of rats was administered the same BPC but not exposed to NS.

## 4. Conclusions

A comparative assessment of adverse bioactivity of virtually equidimensional gold and silver nanoparticles administered to rats at equal mass doses either intratracheally (with a single instillation) or intraperitoneally (with repeated injections) has demonstrated that:

- Pulmonary deposition of both nanometals, but nanosilver (NS) more than nanogold (NG), evokes significant recruitment of phagocytic cells, especially of neutrophile leukocytes (NL) to the free surface of lower airways which we consider to be a predominantly defensive response.
- The count ratio of NLs to alveolar macrophages (AMs) in the bronchoalveolar lavage fluid in both cases is significantly increased as compared with its control value, but more so under exposure to NS. This difference in the NL/AM index may be regarded as a circumstantial but reliable evidence for a higher cytotoxicity of the nanosilver’s.
- Within both AMs and NLs there are a lot of nanoparticles (presumably of NS or NG), and there is TEM and sc-AFM evidence of the part played by active endocytosis, rather by diffusion only, in nanoparticle internalization, this inference being in agreement with that previously made based on a similar experiment with nano-iron oxide.
- There are marked differences between NS and NG as concerns the intracellular distribution of particles within both AM and NL, the most important ones being the ability of NG, but not of NS, to penetrate into nuclei and more marked affinity of NS to mitochondria with more expressed damage to these organelles.
- There is a less marked but still noticeable difference between the same nanometals as concerns their bio-distribution on organism level after repeat i.p. injections: specifically, NG seems to have a somewhat higher affinity to liver and spleen, but much lesser affinity to kidneys as compared with NS, both differences being presumably due to a lower solubility of NG.
- Judging by the degree of the genomic DNA fragmentation (the RAPD test) in cells of many tissues, NG, and much more so NS exert genotoxic action *in vivo* even if the dosage is close to the LOAEL in respect to systemic toxicity assessed by a great number of functional and biochemical indices. Thus, we maintain that it is just genotoxicity that should be considered the limiting health risk under exposure to both nanometals. Whether it may permit one to predict carcinogenic risk is a question demanding further research.
- The difference between the same nanometals as concerns their subchronic systemic toxicity is far less marked, yet NS is more toxic than NG, notwithstanding the latter’s higher accumulation in the RES-abundant organs.

Oral administration of a bioprotective complex comprising pectin, some vitamins, glutamate, glycine, acetyl-cysteine, calcium, selenium, and a fish oil preparation rich in omega-3 PUFA attenuates the toxicity and, especially, the genotoxicity of NS.

## Figures and Tables

**Table 1:** Number of cells in the bronchoaveolar lavage fluid (BALF) 24 h after the intratracheal instillation of suspension of gold or silver particles to rats at a dose of 0.2 mg per rat (*x* ± *S_x_*).

Substance administered	Number of cells * (×10^6^)			NL/AM

	Total	Neutrophil leukocytes (NL)	Alveolar macrophages (AM)	
Based on the results of an earlier experiment [5] with particles of 3.8 nm gold and 3.6 nm silver particles				

Nanosilver	2.78 ± 0.58 *	2.13 ± 0.52 *	0.64 ± 0.07	3.17 ± 0.49 *
Nanogold	2.37 ± 0.49 *	1.45 ± 0.22 *	0.93 ± 0.14	1.67 ± 0.20 *•
Water (controls)	0.99 ± 0.17	0.06 ± 0.01	0.93 ± 0.17	0.05 ± 0.01

Based on the results of this experiment with 50 nm gold and 49 nm or 1.1 μm silver particles				

Nanosilver	4.25 ± 0.77 ∘	2.99 ± 0.71 *∘	1.16 ± 0.14	2.47 ± 0.33 *∘
Microsilver	1.99 ± 0.25	0.73 ± 0.15 *	1.24 ± 0.19	0.66 ± 0.13 *
Nanogold	2.30 ± 0.93 •	0.63 ± 0.15 *•	0.94 ± 0.09	0.63 ± 0.13 *•
Water (controls)	1.41 ± 0.33	0.13 ± 0.04	0.89 ± 0.18	0.14 ± 0.023

Note: statistically significant difference

* from control group;

• from nanosilver group;

∘ from microsilver group (*p* < 0.05 by Student’s *t*-test).

**Table 2:** Indices for the condition of the organism of rats exposed to subchronic i.p. iNjection with particles of nanosilver and nanogold (*X* ± s.e.).

Indices	Groups of rats administered		

	Nanogold (NG)	Nanosilver (NS)	Controls
Initial body mass, g	197.1 ± 2.41	197.1 ± 2.25	196.25 ± 2.62
Body mass after period of injections, g	235.83 ± 4.30	232.08 ± 4.71	234.5 ± 5.38
Temporal summation of sub-threshold impulses, second	15.9 ± 0.71	15.28 ± 1.27	13.5 ± 1.04
Number of head-dips into holes during 3 min	7.36 ± 0.66	7.33 ± 1.11	6.0 ± 0.89
Hemoglobin, g/L	134.6 ± 2.6 *	139.9 ± 3.6	147.9 ± 4.0
Erythrocytes, 10^12^ g/L	4.04 ± 0.08 *	3.87 ± 0.13 *	4.34 ± 0.04
Color index	1.69 ± 0.03	1.86 ± 0.089	1.73 ± 0.06
Reticulocytes, ‰	22.67 ± 4.15	31.67 ± 3.25	27.17 ± 5.45
Lymphocytes, %	49.0 ± 3.04	50.9 ± 2.42	46.8 ± 2.94
Segmented neutrophils, %	32.0 ± 2.6	32.75 ± 2.42	38.9 ± 2.99
Band neutrophils,%	3.08 ± 0.5	2.84 ± 0.53	2.5 ± 0.28
Monocytes, %	9.08 ± 0.74 *	7.5 ± 0.64	5.6 ± 0.77
Eosinophils, %	6.1 ± 0.8	5.8 ± 0.9	6.3 ± 0.7
Basophils, %	0.67 ± 0.28	0.5 ± 0.15	0.73 ± 0.23
Total protein in blood serum, g/L	72.96 ± 1.77	75.4 ± 2.5	74.0 ± 1.6
Albumins in blood serum, g/L	43.5 ± 1.12	41.44 ± 1.04	43.1 ± 1.68
Globulins in blood serum, g/L	29.45 ± 1.35	33.92 ± 2.49	31.6 ± 1.80
A/G index	1.51 ± 0.08	1.28 ± 0.08	1.39 ± 0.10
SDH activity, number of formasan granules in 50 lymphocytes	666.17 ± 8.09 *	679.9 ± 12.4 *	805.33 ± 12.6
ALT activity in blood serum, mmol/h *L	0.15 ± 0.02	0.20 ± 0.025	0.19 ± 0.02
AST activity in blood serum, mmol/h *L	0.26 ± 0.02	0.3 ± 0.02	0.25 ± 0.016
De Ritis coefficient	2.29 ± 0.43	1.73 ± 0.26	1.41 ± 0.19
Catalase in blood serum, μmol/L	1.31 ± 0.16	1.22 ± 0.19	1.14 ± 0.23
MDA in blood serum, nmol/L	5.38 ± 0.31 ∘	6.23 ± 0.14	5.84 ± 0.22
Ceruloplasmin in blood serum, mg %	126.15 ± 11.7 ∘	78.75 ± 8.6 *	164.5 ± 20.0
Bilirubin in blood serum, μmol/L	1.4 ± 0.07	1.52 ± 0.12	1.58 ± 0.09
γ—glutamintransferase, units/L	1.8 ± 0.29	2.72 ± 0.65	3.25 ± 0.8
Alkaline phosphatase in blood serum, nmol/(s*L)	86.7 ± 11.9	119.6 ± 17.4	92.54 ± 13.4
Creatinine in blood serum, μmol/L	35.2 ± 1.43	35.54 ± 1.5	33.2 ± 1.2
Daily volume of urine, mL	39.6 ± 5.1	44.4 ± 5.63	49.8 ± 5.01
Urine acidity, pH units	7.31 ± 0.4	7.25 ± 0.2	7.88 ± 0.3
Specific weight of urine	1.014 ± 0.0008	1.014 ± 0.0006	1.015 ± 0
Creatinine in urine, mol/L	0.84 ± 0.07	0.84 ± 0.09	0.7 ± 0.05
Coproporphyrin in urine, nM/L	54.4 ± 15.05	54.4 ± 7.73	44.0 ± 7.4
δ—ALK in urine, μmol/л	6.0 ± 0.98	7.2 ± 0.49	7.6 ± 0.58
Hydroxyproline in urine, μmol/day	0.50 ± 0.09	0.74 ± 0.11	0.90 ± 0.2
Liver mass, g	3.41 ± 0.09	3.35 ± 0.09	3.31 ± 0.11
Kidney mass, g	0.615 ± 0.015 *	0.63 ± 0.015	0.66 ± 0.013
Spleen mass, g	0.37 ± 0.02	0.44 ± 0.04	0.41 ± 0.02

Note:

* the change is statistically significant compared with the controls;

∘ the change is statistically significant compared with the NS group (*p* < 0.05 by Student’s *t*-test).

**Table 3:** Some morphometric indices of the cell structure of rat liver and spleen (*x* ± s.e.).

Indices	Control	Nano-Au	Nano-Ag	Nano-Ag with background administration of BPC
Number of akaryotic hepatocytes per 100 liver cells	17.6 ± 0.6	19.1 ± 0.2	18.5 ± 1.3	13.0 ± 1.0 *•
Number of binucleate hepatocytes per 100 liver cells	5.9 ± 0.8	8.7 ± 0.6 *	7.8 ± 0.6	12.0 ± 1.5 *•
Number of Kupffer cells per 100 liver cells	16.5 ± 0.5	25.3 ± 0.6 *	25.0 ± 0.8 *	20.0 ± 0.6 *•
Weighted average particle load of Kupffer cells, score #	0	0.35 ± 0.08 •	0.91 ± 0.7	0.51 ± 0.09 •
White to red pulp ratio of spleen †	0.59 ± 0.036	0.37 ± 0.028 *	0.37 ± 0.035 *	0.59 ± 0.086 •

Note: statistically significant difference

* from control group;

• from the (Nano-Ag) group (*p* < 0.05; by Student’s *t*-test);

# The particle burden of a cell is visually estimated as a score of points from 0 to 4. The weighted average index is calculated allowing for the percentage ratio between cells given different scores (the total number of scored cells—100);

† Measured with the help of a planimetric grid.

**Table 4:** Coefficients of the genomic DNA fragmentation (Cfr) in rats exposed to subchronic administration of nanosilver particles with and without protection by bioprotective complex (BPC), or of nanogold particles (based on the results of RAPD-test), *X* ± s.e.

Group of rats exposed to	Tissues					

	Liver	Bone marrow	Spleen	Kidney	Nucleated cells of peripheral blood	Skeletal muscle
Water (controls)	0.399 ± 0.001	0.385 ± 0.003	0.379 ± 0.002	0.385 ± 0.003	0.383 ± 0.001	0.352 ± 0.002
Nano-gold-(NG)	0.392 ± 0.010∘	0.412 ± 0.014 *	0.397 ± 0.008∘	0.422 ± 0.009 *	0.403 ± 0.018	0.340 ± 0.010
Nano-silver (NS)	0.461 ± 0.002 *	0.455 ± 0.032 *	0.462 ± 0.001 *	0.423 ± 0.008 *	0.413 ± 0.012 *	0.356 ± 0.009
NS + BPC	0.408 ± 0.011 +	0.373 ± 0.003 *+	0.419 ± 0.003 *+	0.407 ± 0.006 *+	0.390 ± 0.007	0.331 ± 0.015 *

Note: statistically significant difference

* from the control group;

+ between the group given NS together with BPC and the group given NS only;

∘ between the group receiving NG and the group receiving NS (*p* < 0.05 by Student’s *t*-test).

**Table 5:** Gold or silver contents of the liver, spleen and kidneys (mg/g of freeze-dried tissue) of rats after repeat intraperitoneal injections of corresponding nanometals (*X* ± s.e.).

Group of rats given:	Tissues		

	Liver	Spleen	Kidney
Water (controls)	0.0017 ± 0.0003	0.02 ± 0.007+	0.002 ± 0.0007
Nanogold (NG)	0.20 ± 0.02 *∘	0.50 ± 0.1 *+	0.010 ± 0.001 *∘+
Nanosilver (NS)	0.12 ± 0.01 *	0.40 ± 0.04 *+	0.26 ± 0.09 *
NS + BPC	0.11 ± 0.01 *	0.43 ± 0.04 *+	0.23 ± 0.05 *+

Note: statistically significant difference:

* from the control group;

∘ from the group receiving NS;

+ from the metal content of the liver (*p* < 0.05 by Student’s *t*-test).

**Table 6:** Indices for the condition of the organism of rats exposed to subchronic instillation with particles of nanosilver with and without protection with BPC (*X* ± s.e.).

Indices	Groups of rat receiving			

	Nanosilver (NS)	NS + BPC	BPC	Controls
Initial body mass, g	197.1 ± 2.25	197.1 ± 2.41	196.7 ± 2.34	196.25 ± 2.62
Body mass after period of injections, g	232.08 ± 4.71	233.50 ± 4.90	235.50 ± 3.94	234.5 ± 5.38
Temporal summation of sub-threshold impulses, second	15.28 ± 1.27	15.45 ± 0.96	12.85 ± 1.02	13.5 ± 1.04
Number of head-dips into holes during 3 min	7.33 ± 1.11	7.58 ± 1.09	6.08 ± 1.21	6.0 ± 0.89
Haemoglobin, g/L	139.9 ± 3.6	143.0 ± 4.0	141.9 ± 2.8	147.9 ± 4.0
Erythrocytes, 10^12^ g/L	3.87 ± 0.13 *•	4.25 ± 0.04	4.04 ± 0.09	4.34 ± 0.04
Colour index	1.86 ± 0.089	1.71 ± 0.04	1.78 ± 0.04	1.73 ± 0.06
Reticulocytes, ‰	31.67 ± 3.25	27.25 ± 2.67	29.67 ± 3.84	27.17 ± 5.45
Lymphocytes, %	50.9 ± 2.42	47.5 ± 3.15	48.5 ± 3.85	46.8 ± 2.94
Segmented neutrophils, %	32.75 ± 2.42	34.4 ± 3.01	33.7 ± 3.3	38.9 ± 2.99
Band neutrophils,%	2.84 ± 0.53	2.67 ± 0.48	3.2 ± 0.44	2.5 ± 0.28
Monocytes, %	7.5 ± 0.64	8.25 ± 1.15	6.3 ± 0.8	5.6 ± 0.77
Eosinophils, %	5.8 ± 0.9	7.3 ± 1.3	7.7 ± 1.6	6.3 ± 0.7
Basophils, %	0.5 ± 0.15	0.75 ± 0.25	0.6 ± 0.27	0.73 ± 0.23
Total protein in blood serum, g/L	75.4 ± 2.5	73.7 ± 1.95	79.96 ± 2.85	74.0 ± 1.6
Albumins in blood serum, g/L	41.44 ± 1.04	42.95 ± 0.9	45.92 ± 0.9	43.1 ± 1.68
Globulins in blood serum, g/L	33.92 ± 2.49	30.82 ± 1.81	34.1 ± 2.26	31.6 ± 1.80
A/G index	1.28 ± 0.08	1.46 ± 0.12	1.39 ± 0.09	1.39 ± 0.10
SDH activity, number of formasan granules in 50 lymphocytes	679.9 ± 12.4 *•	827.8 ± 22.1	834.1 ± 11.2	805.33 ± 12.6
ALT activity in blood serum, mmol/h*L	0.20 ± 0.025	0.21 ± 0.02	0.16 ± 0.026	0.19 ± 0.02
AST activity in blood serum, mmol/h*L	0.3 ± 0.02	0.25 ± 0.02	0.26 ± 0.02	0.25 ± 0.016
De Ritis coefficient	1.73 ± 0.26	1.47 ± 0.24	2.31 ± 0.62	1.41 ± 0.19
Catalase in blood serum, μmol/L	1.22 ± 0.19	1.23 ± 0.22	1.43 ± 0.20	1.14 ± 0.23
MDA in blood serum, nmol/l	6.23 ± 0.14	5.72 ± 0.25	5.78 ± 0.21	5.84 ± 0.22
Ceruloplasmin in blood serum, mg %	78.75 ± 8.6 *	72.62 ± 6.3 *	149.72 ± 12.3	164.5 ± 20.0
Bilirubin in blood serum, μmol/L	1.52 ± 0.12	1.52 ± 0.08	1.47 ± 0.11	1.58 ± 0.09
γ—glutamintransferase, units/L	2.72 ± 0.65	2.52 ± 0.37	2.57 ± 0.8	3.25 ± 0.8
Alkaline phosphatase in blood serum, nmol/(s*L)	119.6 ± 17.4	131.5 ± 11.9 *	108.9 ± 18.4	92.54 ± 13.4
Creatinine in blood serum, μmol/L	35.54 ± 1.5	33.5 ± 1.4	35.7 ± 1.52	33.2 ± 1.2
Daily volume of urine, mL	44.4 ± 5.63	52.3 ± 5.4	41.6 ± 4.76	49.8 ± 5.01
Urine acidity, pH units	7.25 ± 0.2	7.38 ± 0.2	7.58 ± 0.3	7.88 ± 0.3
Specific weight of urine	1.014 ± 0.0006	1.015 ± 0	1.014 ± 0.0007	1.015 ± 0
Creatinine in urine, mol/L	0.84 ± 0.09	0.725 ± 0.056	0.76 ± 0.05	0.7 ± 0.05
Coproporphyrin in urine, nM/L	54.4 ± 7.73	40.0 ± 5.5	40.8 ± 8.36	44.0 ± 7.4
δ—ALK in urine, μmol/л	7.2 ± 0.49 •	5.7 ± 0.45	7.7 ± 0.45	7.6 ± 0.58
Hydroxyproline in urine, μmol/day	0.74 ± 0.11	0.70 ± 0.2	1.03 ± 0.4	0.90 ± 0.2
Liver mass, g	3.35 ± 0.09	3.58 ± 0.04	3.36 ± 0.1	3.31 ± 0.11
Kidney mass, g	0.63 ± 0.015	0.061 ± 0.017 *	0.66 ± 0.023	0.66 ± 0.013
Spleen mass, g	0.44 ± 0.04	0.44 ± 0.03	0.39 ± 0.03	0.41 ± 0.02

Note: statistically significant difference

* from the controls;

• from the NS + BPC group (*p* < 0.05 by Student’s *t*-test).

**Table 7:** Doses and the mode of administration of the bioprotectors tested in our experiment.

Bioprotectors	Estimated dosage and the mode of administration
Sodium glutamate	800 mg/kg (as a 1.5% drink instead of water)
Apple pectin	1 g/kg (added to the fodder)
“Complivit-Se” ( the source of vitamins, Se and Cu)	4 mcg per rat (added to the fodder)
“Complivit–Ca” (the source of vitamins and Ca)	160 mg per rat (added to the fodder)
Glycine	12 mg per rat (added to the fodder)
Acetyl-cysteine	30 mg per rat (added to the fodder)
“Eicosavitol” (the source of omega 3 PUFA)	1 mL per rat (by gavage)
